# Real‐time specific absorption rate supervision for a 32‐channel RF transmit system with virtual observation points

**DOI:** 10.1002/mrm.30643

**Published:** 2025-07-25

**Authors:** Thomas M. Fiedler, Stephan Orzada, Johannes A. Grimm, Bottyan Batkai, Stefan Dinkelacker, Fabian J. Kratzer, Christoph Klein, Markus W. May, Falk Mayer, Luisa Schweins, Mark E. Ladd

**Affiliations:** ^1^ Medical Physics in Radiology German Cancer Research Center (DKFZ) Heidelberg Germany; ^2^ Erwin L. Hahn Institute for MRI University Duisburg‐Essen Essen Germany; ^3^ Faculty of Physics and Astronomy Heidelberg University Heidelberg Germany; ^4^ Medical Image Computing German Cancer Research Center (DKFZ) Heidelberg Germany; ^5^ High‐Field and Hybrid MR Imaging University Hospital Essen Essen Germany; ^6^ Faculty of Medicine Heidelberg University Heidelberg Germany

**Keywords:** GPU acceleration, local SAR, safety supervision, UHF MRI, VOPs

## Abstract

**Purpose:**

Real‐time supervision is a crucial element of an RF parallel transmit (pTx) system to supervise safety of the subject during MR imaging and to utilize the full potential of the RF array. However, the computational demand for the specific absorption rate (SAR) calculation scales much greater than linearly with the number of RF channels. Furthermore, a high number of virtual observation points (VOPs) for the local SAR supervision is preferable to reduce the SAR overestimation during the VOP compression, increasing the computational demand further. An RF transmit supervision system for a 32‐channel pTx system including local SAR calculation with a high number of VOPs was developed.

**Methods:**

The system includes 64 digitizer channels to measure the real and imaginary parts of 32 transmit channels. To handle the high computational demand, local SAR calculation is performed on a graphics processing unit (GPU). SAR is averaged for 10 s and 6 min. The system operates independently of the MR system and shuts down the RF power amplifiers (RFPAs) if a SAR limit is exceeded, or the supervision system is interrupted.

**Results:**

The presented system is able to monitor 32 transmit channels and perform real‐time SAR calculation with up to 165 000 VOPs. When using only 16 or 8 channels, the number of VOPs increases to 730 000 and 2 300 000, respectively.

**Conclusion:**

In this work, we present a real‐time RF supervision system designed to monitor a 32‐channel pTx systems including the relative phases of each channel and to perform the local SAR calculation based on VOPs from numerical simulations.

## 1 INTRODUCTION

In ultra‐high‐field (UHF) MRI, multi‐channel RF transmit antenna arrays and parallel transmit (pTx) techniques are used to influence the *B*_1_^+^ distribution and optimize the spin excitation field in a region of interest by superimposing the antenna fields with different amplitudes and phases. Not only the magnetic, but of course the electric field varies with the time‐dependent amplitudes and phases, leading to a different distribution of the specific absorption rate (SAR) for each set of complex shim weightings.^1^ Thus, SAR is now a function of the complex excitation vector s(t) containing all amplitudes and phases, and not simply a function of total input power as in single‐channel transmit systems. Human tissue absorbs energy then exposed to electric fields. The energy absorption is proportional to the conductivity σ>0 in S/m of the tissue and transformed into a heat input that must be limited to prevent tissue damage.

In pTx, SAR is thus pulse and time dependent:

$$\mathrm{SAR}\left(r,t\right)=\frac{1}{T}\int_{0}^{T}\left(\frac{1}{V}\int_{V}\frac{\sigma \left(r\right)}{2\rho \left(r\right)}{\left\|\vec{E}\left(r,t\right)\right\|}^{2}\mathrm{dV}\right)\mathrm{dt}\;\text{with}\;\vec{E}\left(r,t\right)=\sum_{c=1}^{N_{\mathrm{CH}}}{\vec{E}}_{c}\left(r\right)\cdot s_{c}\left(t\right)$$

with the electrical conductivity σ in S/m, the specific tissue density ρ in kg/m^3^, the magnitude of the electric field $\vec{E}$ in V/m, $N_{\mathrm{CH}}$ the number of transmit channels and the complex voltage of the transmit signal s(t) in V.

With the SAR matrix formalism, a separation of the spatial and temporal dependencies can be performed. The SAR matrices contain the spatial dependencies, including an electric field normalized to the transmit signal^2^:

$$Q\left(r\right)=\frac{1}{V}\int_{V}\frac{\sigma \left(r\right)}{2\rho \left(r\right)}{\vec{E}}^{H}\left(r\right)\cdot \vec{E}\left(r\right)\mathrm{dV}.$$

Together with the temporal dependencies, SAR can be calculated for each time point during the MR experiment with the quadratic form^3^, ^4^:

$$\mathrm{SAR}\left(r,t\right)=\frac{1}{∆T}\cdot \int_{∆t}{\vec{s}}^{H}\left(t\right)\cdot Q\left(r\right)\cdot \vec{s}\left(t\right)\;\mathrm{dt}.$$

The SAR matrices are of size $N_{\mathrm{CH}}\times N_{\mathrm{CH}}$.

**FIGURE 1:**
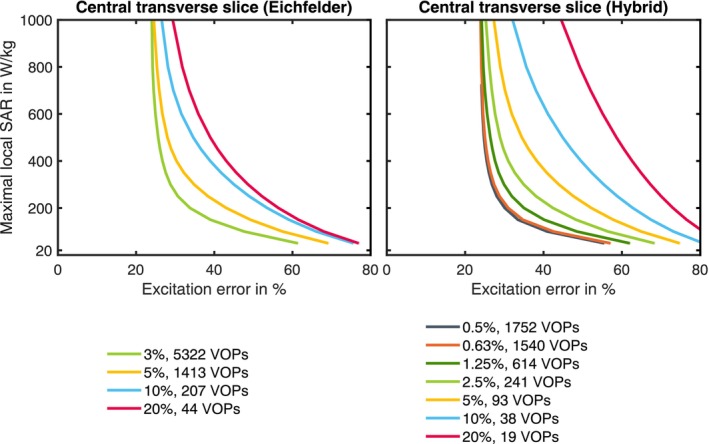
Demonstration of the effect of the compression overestimation and the number of VOPs, which is related to the SAR overestimation on the RF shim performance. Two different algorithms from Eichfelder and Gebhard^6^ and Orzada et al.^9^ were used. The field optimization was performed in a central transverse slice of the body (liver‐kidney region) for the 32‐channel body array at 7T with a constraint of 1200 W/channel. SAR is given for a continuous‐wave signal; the SAR limit determines the maximum duty‐cycle of the sequence.

In numerical simulations for RF antennas with anatomical body models, a local SAR matrix is defined per mesh cell. As anatomical body models can consist of several million mesh cells, local SAR supervision is a major obstacle in the safety supervision of pTx systems.^5^ To reduce the number of matrices for real‐time supervision, Eichfelder and Gebhard introduced the concept of virtual observation points (VOPs),^6^ where a compressed set of SAR matrices is computed from the simulation data, reducing the number of matrices to supervise from several million to a few hundred. The compression algorithms based on this approach incorporate an overestimation factor that is defined as a percentage of the worst‐case SAR. A high overestimation factor results in a low number of VOPs but a high SAR overestimation, which reduces the imaging performance of the RF array, as the supervision estimates a higher SAR compared to the actual SAR due to the VOP overestimation. As shown previously, the relative overestimation for a specific RF shim can easily reach several hundred percent, as the worst‐case SAR is the maximum SAR value achievable by any RF shim.^5^ Thus, a low overestimation factor is preferable, which in turn leads to a high number of VOPs. Further research on VOP compression has therefore focused on the reduction of the number of VOPs and the reduction of the overestimation.^7^, ^8^, ^9^ The effect of the SAR overestimation is shown in Figure 1 for a 32‐channel body array in the form of L‐curves.

To supervise safety of the subjects during imaging, the transmitted RF power must be monitored in real time by an independent supervision system that stops the transmission of the RF signal if one of the limits defined in the IEC standard is exceeded.^10^, ^11^, ^12^ The IEC guidelines define limits for the local SAR, where SAR is calculated and averaged over any 10 g volume in the body, and for global SAR aspects (whole body, partial body, and head).^13^ For both, the averaging time is 6 min and SAR limits over any 10 s period shall not exceed two times the stated values. According to the current edition of the International Electrotechnical Commission (IEC) guidelines, volume transmit coils shall be controlled with conservative estimates of whole‐body SAR, head SAR, and at least one of the following: partial body SAR or local SAR. Local transmit coils shall be controlled by conservative estimates of local SAR and whole‐body SAR. Multi‐channel transmit coils can have attributes of both local and volume RF transmit coils, and the appropriate control of SAR depends on the use of the coil.

Today, most UHF MR sites use an 8‐channel pTx system with some sites also using 16 channels. Previously, we presented a 32‐channel RF transmit add‐on system including an integrated body coil antenna array for imaging at 7T.^14^, ^15^ Although this array exposes a large part of the human body, we demonstrated that the local SAR limit is always reached before the whole‐body limit,^16^ highlighting the need for the local SAR supervision. The global SAR aspects (whole body, partial body, head) can be represented by a single SAR matrix and added to the supervision, which increases the computational load only minorly.

The computational demand for the SAR calculation also scales with the number of RF channels. A 32‐channel system requires 16‐times more floating‐point operations per SAR matrix compared to an 8‐channel system (2048 multiplications and 1024 additions versus 128 multiplications and 64 additions per matrix). This can be a limiting factor for calculating SAR in real time. Graphics processing units (GPUs) can be used for parallel computing of the SAR calculation.

In this work, we present a safety supervision system designed to address two challenges: first, to monitor 32 RF transmit channels including their relative phases, resulting in a total of 64 data channels; second, to perform the local SAR calculation within the data acquisition time to ensure real‐time supervision, using a high number of VOPs to reduce the SAR overestimation.

## 2 METHODS

### 2.1 Data acquisition

Eight digitizer cards (Teledyne ADQ14‐4C, 1 GSPS sampling rate, 14‐bit vertical resolution, max. 1.8 V_pp_ input range, four digital down converters [DDC] per card) are used for sampling of the RF signal. Each DDC has one channel for the real and one for the imaginary part, resulting in a total of 64 channels. The digitizers are connected to a custom‐built workstation (Intel Core i7‐10700K, 32 GB DDR4, running on Ubuntu 24.04) via a Gen2‐x8‐PCIe connection (Adlink PCIe‐PXIe‐8638). For synchronous operation, the digitizer cards are set to the PCIe reference clock and phase‐synchronized upon restart with an external reference signal (297.15 MHz, 0.55 V_pp_) that was validated with a E5071C network analyzer (Agilent Technologies, Santa Clara, California, USA). The sampling rate is set to 1.953 MHz, resulting in a data stream of sampling frequency × 64 channels × 2 bytes (16 bits) = 0.25 Gbytes/s. The cards are factory‐calibrated with a conversion factor from digital value to voltage for each DDC.

The code for the data acquisition on the workstation is written in C++. The data stream of the 64 data channels from the DDCs (real and imaginary part) is combined to 32 complex signals, and the recorded data are split into data blocks consisting of 8192 16‐bit data samples (record time per block ∆ *T* = 4.19 ms).

Thirty‐two directional couplers (DiCo) are placed in the transmit path of the RF signal and connected via coaxial cables with the digitizers to measure the forward transmit RF signal.^14^, ^17^ The output of the DiCos was adjusted with attenuators to match the input range of the digitizers. The coupling factor of the DiCos and attenuation of the cables were measured together to define a scaling factor for each channel, which establishes the position of the DiCo input as the reference for the SAR calculation. Consequently, all cables from the output of the DiCos to the RF antenna should be included in the simulation model used to generate the VOPs.

**FIGURE 2:**
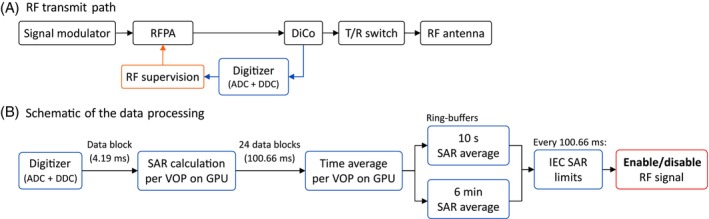
(A) Simplified overview of the RF transmit path (black arrows) from the signal modulators to the RF antenna, the signal flow from the DiCo to the RF supervision (blue arrows), and the control (shutdown and heartbeat) signals to the RFPA (orange arrow). (B) Simplified overview of the signal processing and SAR averaging with ring‐buffers for 10 s and 6 min averaged SAR.

A simplified schematic overview of the RF transmit path is shown in Figure 2.

### 2.2 SAR calculation and time averaging

**FIGURE 3:**
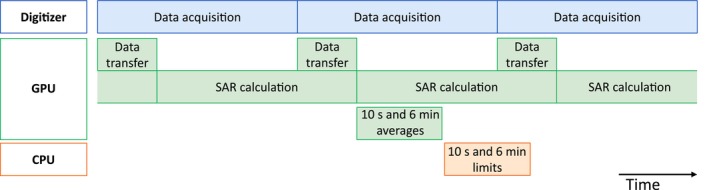
Timing diagram of the continuous data acquisition and SAR evaluation. Blue: Data acquisition on the digitizer cards. Each data block contains 8192 samples recorded in 4.19 ms. Green: Data transfer to the GPU and local SAR calculation. The time averaging on the GPU is repeated after every 24 recorded data blocks (100.66 ms). Orange: Evaluation of the maximum average values regarding the SAR limits on the host CPU.

Each complete recorded data block is sent to a GPU (NVIDIA RTX 3090, 24 GB GDDR6, 35.6 TFLOPS FP32 [NVIDIA Corp., Santa Clara, CA, USA]) on the afore‐mentioned workstation for SAR calculation. The data transfer takes place in parallel to the SAR calculation (Figure 3), so that the SAR calculations run continuously with maximum GPU utilization. The symmetric VOP matrices from numerical simulations are provided as a HDF5 file in upper‐packed storage mode with single‐precision floating‐point format (FP32). The VOPs are copied once to the GPU during startup.

Local SAR calculation is performed using the quadratic form for each data sample (Algorithm A):

$$\mathrm{SAR}\left(t,v\right)={\vec{s}}^{H}\left(t\right)\cdot A\left(v\right)\cdot \vec{s}\left(t\right)\forall v$$

where $A\in {\mathbb{C}}^{N_{v}\times N_{\mathrm{CH}}\times N_{\mathrm{CH}}}$ are the VOP matrices, $s\in {\mathbb{C}}^{N_{\mathrm{CH}}\times 1}$ is the complex RF signal, $N_{\mathrm{CH}}$ the number of RF channels, and $N_{v}$ the number of VOP matrices. The vector–matrix–vector calculation for each matrix is performed in parallel on a CUDA block. Next, the sum of all SAR values over the data samples per VOP is performed on the GPU.

A second algorithm (Algorithm B) is based on the concept by Graesslin et al.^18^ This algorithm computes first a complex matrix from the signal vector, averaged over the signal samples. SAR is then calculated via a matrix–matrix multiplication for each VOP matrix:

$$s_{\mathrm{avg}}=\bar{{\vec{s}}^{H}\left(t\right)\cdot \vec{s}\left(t\right)}$$

$$\mathrm{SAR}\left(v\right)=\sum_{i=1}^{N_{\mathrm{CH}}}\sum_{j=1}^{N_{\mathrm{CH}}}A_{\mathrm{ij}}\left(v\right)\cdot s_{\mathrm{avg},\mathrm{ij}}\;\forall v.$$

Since the VOP matrices are Hermitian symmetric ($A_{\mathrm{ij}}=A_{ji}*$), only the triangular part needs to be computed. For Algorithm B, the matrix–matrix multiplication becomes:

∑i=1NCHAii·savg,ii+2·∑i=1NCH∑j=i+1NCHReAij·savg,ij

The SAR calculation is performed with three sequential CUDA kernels. The first kernel transforms the signal in complex matrix form with one signal sample per thread block and a 2D thread dimension (32, 32). The second kernel calculates the mean value per matrix element with one matrix element (ij) per thread block and 256 threads per block using a parallel binary reduction (pairwise summation) with shared memory. The third kernel performs the matrix–matrix multiplication for the triangular matrix with one VOP matrix per thread block. The number of VOP matrices was adjusted for maximum GPU utilization. The SAR calculation is performed within the 4.19 ms record time for the subsequent data block (Figure 3). The SAR of 24 data blocks (100.66 ms) is time‐averaged per VOP and added to a ring buffer containing 3600 entries. The ring buffer is evaluated every 100.66 ms for the 6 min averaged SAR. Furthermore, the ring buffer is evaluated in reverse order (backwards in time) in order to additionally obtain the 10 s average from the most recent 100 entries. The maximum values for both time intervals are transferred to the CPU every 100.66 ms and evaluated regarding the IEC SAR limits. If the local SAR limit for 10 s or 6 min is exceeded, the RF signal is stopped by setting a TTL shutdown signal to low, which switches off the bias voltage of the two final stages of the RF power amplifiers (RFPAs) as well as the drain voltage of the final stage.^19^ The time interval between the activation of the TTL shutdown signal and the subsequent cessation of the RF signal was determined. The supervision system continues to monitor the RF signal and will re‐enable the RF signal as soon as the time‐averaged SAR is below the limit. A delay of 5 s before re‐enablement is set after activation of the shutdown signal to prevent the RFPAs from switching off and on rapidly. In addition, the maximum SAR is calculated for each 4.19 ms data block. This enables short pulses with a high SAR to be detected, which would otherwise exceed the 10 s limit before the 100 ms evaluation.

The code for the SAR calculation is written in CUDA (NVIDIA CUDA compiler 11.8). NVIDIA Nsight Systems was used for GPU profiling. For comparison and validation, a central processing unit (CPU) ‐based version of the SAR calculation was added to the code.

To monitor the RFPAs and compare the RF shim settings, the averaged and maximum RF power per channel are displayed after each 1 s time interval.^20^

### 2.3 System supervision

The digitizer cards are configured for continuous streaming and will report an error if the software for the SAR calculation is delayed and the data are not fetched in time.

The supervision software running on the Ubuntu workstation sends an alternating (“alive”) TTL signal every 0.1 s to the RFPAs via the GPIO ports on the digitizer cards. If a signal is not followed up by the next signal within 0.11 s, the RFPAs will switch off within a few μs.

SAR was calculated in advance for each VOP file using the external reference signal (previously used for the phase synchronization). During system startup, SAR is recalculated with the measured reference signal and compared with the stored results for the selected VOP file. If a deviation >2.5% is detected, the system does not continue startup and will not enable the RFPAs. This check verifies that all digitizers are successfully initialized, the VOP file is unaltered, and the reference signal is active and at a predefined level.

### 2.4 Measurement error, validation, and stability

The factory calibration of the digitizer cards and all steps of the supervision software were validated with pre‐defined power levels (−5, 3, 5, 9 dBm) from an HM8134‐3 RF synthesizer (Rohde & Schwarz GmbH & Co. KG, Munich, Germany). The output of the synthesizer including a 30 cm connection cable was measured with a LadyBug LB479A power sensor (LadyBug Technologies, LLC., Boise, ID, USA).

The SAR calculation and time averaging were cross‐checked with an implementation in Matlab (The Mathworks, Inc., Natick, MA, USA), which is not suitable for real‐time monitoring. Recorded data sets were evaluated using Matlab regarding power level and waveform.

The system is placed in a climate‐controlled room. The measurement system was continuously active over 14 days and a data set was recorded on the first and last day to evaluate the stability over time. For this, a continuous‐wave signal at 297.15 MHz from the RF synthesizer and an eight‐channel power splitter (Mini‐Circuits ZBSC‐8‐82‐S+), splitting the signal to one channel of each digitizer card, was used. The magnitude of the real and imaginary signal was compared between the data from the first and the last day.

## 3 RESULTS

### 3.1 Data acquisition

The data stream of 64 channels with a sampling frequency of 1.953 MHz in single‐precision format requires a bandwidth of 0.25 Gbyte/s, which is within the maximum of 4 Gbyte/s connection bandwidth.

**FIGURE 4:**
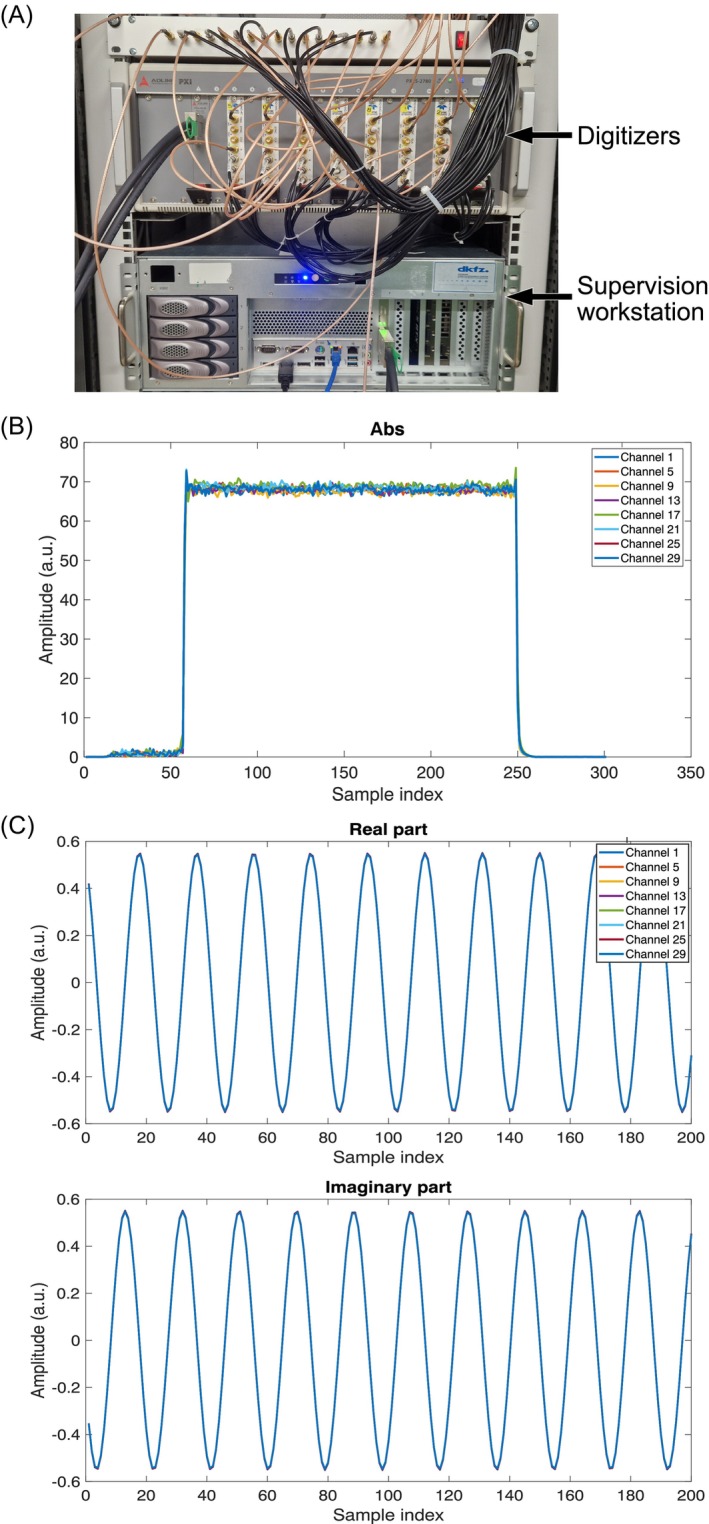
(A) Photograph of the digitizers and the supervision workstation. Different waveforms measured with the first channel of each digitizer card. (B) Absolute value of an RF signal transmitted with the pTx system; requested: 10 ms rect pulse, measured: 195 samples * 5.12E‐7 s (1.953 MHz) = 9.984 ms rect pulse. (C) Real and imaginary part of a continuous signal from the RF synthesizer set to 297.2 MHz. The signal was split with a Mini‐Circuits ZBSC‐8‐82‐S+ eight‐channel power splitter. The mixer frequency of the digitizers was set to the same frequency. The measurement in B shows noise after the unblank signal on the RFPAs before and during transmission of the RF pulse.

Figure 4 shows a representation of different waveforms from the MR system^14^ and the RF synthesizer, all acquired with the selected sampling rate and for the first channel of each digitizer card. Figure 4B shows a rect pulse, demonstrating the correct pulse length and the correct timings. Figure 4C shows a continuous signal from the RF synthesizer showing the correct phase synchronization.

### 3.2 SAR calculation

With the selected GPU, up to 165 000 32‐channel VOPs can be supervised continuously in real time with Algorithm B. In addition, the maximum number of VOPs was evaluated for 8 and 16 channels. Here, the maximum number of VOPs is 2 300 000 for 8 channels and 730 000 VOPs for 16 channels. Algorithm A requires more floating‐point operations^18^ and resulted in a lower number of VOPs, Table 1. The number of VOPs for eight channels with Algorithm B was limited by the GPU memory.

**TABLE 1:** Maximum number of VOPs that can be supervised in real‐time for different numbers of RF channels using the proposed supervision system with one GPU (NVIDIA RTX 3090).

No. of RF channels		8	16	32
Max. number of VOPs	Algorithm A	23 000	4800	1450
	Algorithm B	2 300 000^a^	730 000	165 000

^a^ The eight‐channel configuration was limited by the GPU memory.

Figure 1 shows optimizations of the RF field with different overestimations for the 32‐channel body array and a male body model^21^ with the liver/kidney region in the center. An overestimation of 1.25% with the hybrid algorithm results in 614 VOPs. Evaluating the homogeneity for example with the SAR constraint of 200 W/kg (continuous‐wave signal without duty‐cycle), results in an excitation error of 35%. Increasing the number of VOPs to 1540 reduces the error to 31%. In contrast, reducing the number of VOPs to 20 would increase the excitation error to 73%.

### 3.3 Measurement error, validation, and stability

A deviation in the phase of the reference signal of <0.1° was found.

All digitizer channels were validated with the HM8134‐3 RF synthesizer where a maximum deviation of 3.99% in signal magnitude was found. The deviation per channel was included as a correction factor to the factory calibration. All data are shown in the Supporting Information (Table S1). Continuous measurement over a time‐period of 14 days showed a deviation of 0.12% on the magnitude of the real or imaginary signal.

The shutdown of the RFPAs was tested with different pulse forms and pulse durations. If the 10 s or the 6 min limit is exceeded, the RF signal is immediately deactivated and the user is informed. The time between activation of the TTL shutdown signal and the subsequent cessation of the RF signal is <100 μs.

## 4 DISCUSSION

Real‐time SAR supervision is a crucial element of an RF pTx system to ensure safety of the subject during MR imaging and to terminate RF transmission if one of the limits is exceeded. However, the computational demand for the SAR calculation scales greater then linearly with the number of RF channels. Furthermore, as arrays with a high number of channels tend to have higher worst‐case local SAR values, it is preferable to use a high number of VOPs in the supervision of these arrays to reduce SAR overestimation, which in turn increases the computational demand further but enables the full potential of the RF array to be utilized without imposing unnecessary limitations on the transmit power.

In this work, we present a real‐time RF supervision system designed to monitor the forward transmit power in a 32‐channel pTx system including the relative phases of each channel and to perform the local SAR supervision. To handle the computational demand arising from local SAR calculation, the calculation was performed on a GPU to take advantage of the parallel compute capabilities. As an independent supervision system, this system can monitor any RF transmit array with up to 32 channels in real time using the corresponding VOP file obtained from numerical simulations. These VOP matrices can be defined for local SAR, whole‐body SAR, head SAR, or a combination of all.

In addition, the maximum number of VOPs was evaluated for a reduced configuration of 8 and 16 channels. A direct comparison with the supervision of a commercially available MRI system is however difficult, as detailed information about the SAR calculation is not publicly available and the performance of the SAR supervision heavily depends on the individual hardware of the MR system and varies between different devices. Furthermore, the SAR supervision of some systems is combined with other task, for example, the image reconstruction.

This work demonstrates the capability to handle a high number of matrices in the GPU‐accelerated SAR calculation. While this capability can be used to reduce SAR overestimation, a high number of VOP matrices will lead to significant delays during constrained patient‐specific RF pulse optimizations. This could limit the upper number of VOPs to be used. Alternatively, pre‐calculated optimizations can be used, for example universal pulses.^22^

With advances in the development of more powerful CPUs and GPUs, newer workstations will be able to supervise more RF channels and/or use a higher number of VOPs. Furthermore, the system design is scalable and can be accelerated further by using multiple GPUs, as the SAR calculation can be split and distributed to several GPUs. The calculations in this work were performed on a standard desktop GPU. While it would be possible to monitor a smaller number of VOPs on a workstation/server CPU with high core count, GPUs provide more floating‐point operations per second per unit cost.

Two features can be extrapolated from the presented results: the capability to monitor 64‐ or 128‐channel systems, and the possibility to monitor arrays with the uncompressed set of SAR matrices instead of VOPs, which eliminates the SAR overestimation introduced during the VOP compression completely. Uncompressed monitoring of smaller body regions, for example eight‐channel head arrays, is already possible with current hardware generations.

The presented system monitors only the forward transmitted RF power and neglects the reflected power, which is monitored in some commercial pTx systems. DiCos have one output for the forward and one for the reflected RF power. However, a second set of digitizer cards would be necessary to include the reflected power during the real‐time supervision, and these were the most expensive components of this project. Using only the forward power in the safety supervision leads to an overestimation of SAR and is a more conservative approach. An evaluation for the integrated 32‐channel array shows 4% reflected power. If all possible positions are evaluated and the minimum reflected power has been found, a correction factor can be included in the safety supervision to calculate the accepted power ($P_{\mathrm{acc}}=P_{\text{forward}}-P_{\text{reflected}}$). In the case of only one set of digitizer cards, one option is to check only for defective hardware components, for example, broken RF antennas or cables, by placing an RF switch between the DiCos and digitizers to switch between forward and reflected power, feeding either of these two signals to the digitizers. The reflected power can then be evaluated prior to the MR measurement to test for defective components.

## 5 CONCLUSIONS

This work presents a real‐time local SAR supervision concept for a 32‐channel transmit system that includes amplitude and phase measurements and is designed with GPU‐accelerated SAR calculation using VOP matrices. The system has a scalable design and monitors up to 165 000 VOPs with the current hardware configuration. The high number of VOPs allows the reduction the SAR overestimation and demonstrates the ability to supervise an RF antenna array even with the uncompressed SAR matrices, thereby completely eliminating the overestimation.

## Supporting information


**Table S1.** Validation and calibration of the digitizer channels with pre‐defined power levels (−5, 3, 5, 9 dBm) on an HM8134‐3 RF synthesizer (Rohde & Schwarz GmbH & Co. KG, Munich, Germany). The output of the synthesizer including a 30 cm connection cable was measured with a LadyBug LB479A power sensor (LadyBug Technologies, LLC., Boise, ID, USA).
