# Supplementary material for: Real‐time specific absorption rate supervision for a 32‐channel RF transmit system with virtual observation points
Source: Magn Reson Med. 2025 Jul 25;94(6):2785–93. doi: 10.1002/mrm.30643 (PMC12501689; doi:10.1002/mrm.30643)
Supplement: Supplementary file 1 — Table S1. Validation and calibration of the digitizer channels with pre‐defined power levels (−5, 3, 5, 9 dBm) on an HM8134‐3 RF synthesizer (Rohde & Schwarz GmbH & Co. KG, Munich, Germany). The output of the synthesizer including a 30 cm connection cable was measured with a LadyBug LB479A power sensor (LadyBug Technologies, LLC., Boise, ID, USA). [file MRM-94-2785-s001.pdf]

Supporting Information Table S1:

Validation and calibration of the digitizer channels with pre-defined power levels (-5 dBm, 3 dBm, 5 dBm, 9 dBm) on an HM8134-3 RF synthesizer (Rohde & Schwarz GmbH & Co. KG, Munich, Germany).

The output of the synthesizer including a 30 cm connection cable was measured with a LadyBug LB479A power sensor (LadyBug Technologies, LLC., Boise, ID, USA).

I: Measurement data absolut

				Measurement 1				Measurement 2				Measurement 3			
Reference Signal (Rohde & Schwarz HM8134-3)			dBm	-5 dBm	3 dBm	5 dBm	9 dBm	-5 dBm	3 dBm	5 dBm	9 dBm	-5 dBm	3 dBm	5 dBm	9 dBm
			Vrms	0.1256 V	0.3158 V	0.397 V	0.63 V	0.1256 V	0.3158 V	0.397 V	0.63 V	0.1256 V	0.3158 V	0.397 V	0.63 V
			Vp	0.1218 V	0.446 V	0.5623 V	0.8912 V	0.1218 V	0.446 V	0.5623 V	0.8912 V	0.1218 V	0.446 V	0.5623 V	0.8912 V
			Vpp	0.355 V	0.893 V	1.125 V	1.7825 V	0.355 V	0.893 V	1.125 V	1.7825 V	0.355 V	0.893 V	1,125 V	1.7825 V
			W	0.3162 mW	1.995 mW	3.162 mW	7.943 mW	0.3162 mW	1.995 mW	3.162 mW	7.943 mW	0.3162 mW	1.995 mW	3.162 mW	7.943 mW
Calibration: LadyBug LB479A (including cable)			dBm	-5.273	2.696	4.7530	8.805	-5.283	2.666	4.7250	8.79	-5.263	2.691	4.7490	8.805
			Vrms	0.1218	0.3048	0.3869	0.6161	0.1217	0.3041	0.3853	0.6144	0.1220	0.3048	0.3863	0.6162
			**Vp**	**0.1723**	**0.4311**	**0.5472**	**0.8713**	**0.1720**	**0.4301**	**0.5449**	**0.8689**	**0.1725**	**0.4311**	**0.5463**	**0.8714**
ADQ		Channel	Ch #												
SPD-06465	1	A	1	0.1765	0.4398	0.5560	0.8865	0.1762	0.4395	0.5559	0.8851	0.1765	0.4399	0.5566	0.8867
		B	2	0.1760	0.4391	0.5555	0.8848	0.1759	0.4387	0.5550	0.8837	0.1761	0.4392	0.5557	0.8849
		C	3	0.1753	0.4372	0.5532	0.8809	0.1752	0.4366	0.5523	0.8795	0.1754	0.4375	0.5533	0.8813
		D	4	0.1759	0.4387	0.5550	0.8836	0.1758	0.4382	0.5542	0.8823	0.1759	0.4388	0.5552	0.8840
SPD-06471	2	A	5	0.1774	0.4425	0.5598	0.8914	0.1776	0.4432	0.5606	0.8925	0.1776	0.4427	0.5599	0.8918
		B	6	0.1776	0.4430	0.5605	0.8917	0.1776	0.4429	0.5604	0.8919	0.1777	0.4434	0.5611	0.8931
		C	7	0.1772	0.4419	0.5590	0.8902	0.1772	0.4419	0.5590	0.8900	0.1773	0.4419	0.5592	0.8905
		D	8	0.1774	0.4423	0.5592	0.8906	0.1774	0.4423	0.5596	0.8909	0.1774	0.4426	0.5599	0.8917
SPD-06470	3	A	9	0.1777	0.4431	0.5603	0.8920	0.1777	0.4430	0.5603	0.8923	0.1779	0.4436	0.5611	0.8937
		B	10	0.1772	0.4418	0.5589	0.8899	0.1772	0.4415	0.5585	0.8892	0.1773	0.4424	0.5597	0.8912
		C	11	0.1778	0.4443	0.5605	0.8925	0.1778	0.4433	0.5608	0.8928	0.1779	0.4434	0.5610	0.8936
		D	12	0.1773	0.4422	0.5594	0.8908	0.1774	0.4421	0.5593	0.8906	0.1776	0.4429	0.5604	0.8924
SPD-06469	4	A	13	0.1765	0.4404	0.5571	0.8867	0.1765	0.4402	0.5570	0.8867	0.1768	0.4405	0.5574	0.8879
		B	14	0.1752	0.4370	0.5528	0.8801	0.1753	0.4371	0.5528	0.8800	0.1755	0.4376	0.5536	0.8817
		C	15	0.1760	0.4388	0.5548	0.8835	0.1760	0.4389	0.5553	0.8840	0.1763	0.4393	0.5557	0.8851
		D	16	0.1760	0.4390	0.5550	0.8840	0.1760	0.4389	0.5550	0.8836	0.1761	0.4393	0.5557	0.8850
SPD-06463	5	A	17	0.1673	0.4172	0.5278	0.8403	0.1674	0.4173	0.5280	0.8400	0.1675	0.4176	0.5283	0.8413
		B	18	0.1672	0.4164	0.5266	0.8386	0.1672	0.4168	0.5273	0.8395	0.1673	0.4171	0.5277	0.8403
		C	19	0.1673	0.4173	0.5277	0.8401	0.1674	0.4173	0.5280	0.8405	0.1675	0.4176	0.5284	0.8415
		D	20	0.1666	0.4155	0.5255	0.8365	0.1667	0.4153	0.5255	0.8364	0.1668	0.4162	0.5266	0.8382
SPD-06464	6	A	21	0.1762	0.4393	0.5557	0.8846	0.1763	0.4395	0.5559	0.8850	0.1765	0.4398	0.5564	0.8861
		B	22	0.1760	0.4384	0.5545	0.8830	0.1760	0.4384	0.5545	0.8828	0.1760	0.4387	0.5552	0.8840
		C	23	0.1757	0.4378	0.5539	0.8819	0.1757	0.4383	0.5544	0.8825	0.1759	0.4384	0.5547	0.8833
		D	24	0.1754	0.4370	0.5527	0.8801	0.1754	0.4372	0.5528	0.8802	0.1755	0.4379	0.5540	0.8822
SPD-06466	7	A	25	0.1775	0.4423	0.5592	0.8905	0.1774	0.4425	0.5598	0.8912	0.1775	0.4424	0.5597	0.8913
		B	26	0.1770	0.4416	0.5586	0.8893	0.1771	0.4415	0.5583	0.8889	0.1772	0.4421	0.5593	0.8906
		C	27	0.1771	0.4416	0.5587	0.8896	0.1771	0.4417	0.5588	0.8896	0.1773	0.4422	0.5591	0.8905
		D	28	0.1773	0.4422	0.5594	0.8906	0.1773	0.4420	0.5591	0.8901	0.1775	0.4428	0.5601	0.8918
SPD-06468	8	A	29	0.1773	0.4417	0.5588	0.8899	0.1773	0.4422	0.5594	0.8907	0.1774	0.4421	0.5595	0.8911
		B	30	0.1774	0.4422	0.5592	0.8905	0.1774	0.4423	0.5595	0.8909	0.1776	0.4430	0.5604	0.8925
		C	31	0.1770	0.4415	0.5584	0.8893	0.1772	0.4416	0.5584	0.8891	0.1774	0.4422	0.5592	0.8906
		D	32	0.1770	0.4415	0.5584	0.8892	0.1771	0.4415	0.5583	0.8890	0.1773	0.4420	0.5591	0.8905
			Mean:	0.1755	0.4376	0.5535	0.8814	0.1755	0.4376	0.5536	0.8813	0.1757	0.4380	0.5542	0.8825
			Min:	0.1666	0.4155	0.5255	0.8365	0.1667	0.4153	0.5255	0.8364	0.1668	0.4162	0.5266	0.8382
			Max:	0.1778	0.4443	0.5605	0.8925	0.1778	0.4433	0.5608	0.8928	0.1779	0.4436	0.5611	0.8937

II: Measurement data relativ

				Measurement 1				Measurement 2				Measurement 3			
Reference Signal (Rohde & Schwarz HM8134-3)			dBm	-5 dBm	3 dBm	5 dBm	9 dBm	-5 dBm	3 dBm	5 dBm	9 dBm	-5 dBm	3 dBm	5 dBm	9 dBm
			Vrms	0.1256 V	0.3158 V	0.397 V	0.63 V	0.1256 V	0.3158 V	0.397 V	0.63 V	0.1256 V	0.3158 V	0.397 V	0.63 V
			Vp	0.1218 V	0.446 V	0.5623 V	0.8912 V	0.1218 V	0.446 V	0.5623 V	0.8912 V	0.1218 V	0.446 V	0.5623 V	0.8912 V
			Vpp	0.355 V	0.893 V	1.125 V	1.7825 V	0.355 V	0.893 V	1.125 V	1.7825 V	0.355 V	0.893 V	1,125 V	1.7825 V
			W	0.3162 mW	1.995 mW	3.162 mW	7.943 mW	0.3162 mW	1.995 mW	3.162 mW	7.943 mW	0.3162 mW	1.995 mW	3.162 mW	7.943 mW
Calibration: LadyBug LB479A (including cable)			dBm	-5.273	2.696	4.7530	8.805	-5.283	2.666	4.7250	8.79	-5.263	2.691	4.7490	8.805
			Vrms	0.1218	0.3048	0.3869	0.6161	0.1217	0.3041	0.3853	0.6144	0.1219	0.3046	0.3862	0.6156
			**Vp**	**0.1723**	**0.4311**	**0.5472**	**0.8713**	**0.1720**	**0.4301**	**0.5449**	**0.8689**	**0.1724**	**0.4308**	**0.5462**	**0.8706**
ADQ		Channel	Ch #												
SPD-06465	1	A	1	1.024%	1.020%	1.016%	1.017%	1.024%	1.022%	1.020%	1.019%	1.024%	1.021%	1.019%	1.019%
		B	2	1.022%	1.019%	1.015%	1.015%	1.022%	1.020%	1.019%	1.017%	1.022%	1.020%	1.017%	1.016%
		C	3	1.018%	1.014%	1.011%	1.011%	1.018%	1.015%	1.014%	1.012%	1.017%	1.016%	1.013%	1.012%
		D	4	1.021%	1.018%	1.014%	1.014%	1.022%	1.019%	1.017%	1.015%	1.020%	1.019%	1.017%	1.015%
SPD-06471	2	A	5	1.030%	1.027%	1.023%	1.023%	1.032%	1.031%	1.029%	1.027%	1.030%	1.028%	1.025%	1.024%
		B	6	1.031%	1.028%	1.024%	1.023%	1.032%	1.030%	1.028%	1.026%	1.031%	1.029%	1.027%	1.026%
		C	7	1.029%	1.025%	1.022%	1.022%	1.030%	1.028%	1.026%	1.024%	1.028%	1.026%	1.024%	1.023%
		D	8	1.030%	1.026%	1.022%	1.022%	1.031%	1.028%	1.027%	1.025%	1.029%	1.027%	1.025%	1.024%
SPD-06470	3	A	9	1.032%	1.028%	1.024%	1.024%	1.033%	1.030%	1.028%	1.027%	1.032%	1.030%	1.027%	1.027%
		B	10	1.028%	1.025%	1.021%	1.021%	1.030%	1.027%	1.025%	1.023%	1.028%	1.027%	1.025%	1.024%
		C	11	1.032%	1.031%	1.024%	1.024%	1.033%	1.031%	1.029%	1.028%	1.032%	1.029%	1.027%	1.026%
		D	12	1.029%	1.026%	1.022%	1.022%	1.031%	1.028%	1.026%	1.025%	1.030%	1.028%	1.026%	1.025%
SPD-06469	4	A	13	1.025%	1.022%	1.018%	1.018%	1.026%	1.024%	1.022%	1.020%	1.026%	1.023%	1.021%	1.020%
		B	14	1.017%	1.014%	1.010%	1.010%	1.019%	1.016%	1.015%	1.013%	1.018%	1.016%	1.014%	1.013%
		C	15	1.022%	1.018%	1.014%	1.014%	1.023%	1.021%	1.019%	1.017%	1.023%	1.020%	1.017%	1.017%
		D	16	1.022%	1.018%	1.014%	1.015%	1.023%	1.021%	1.019%	1.017%	1.022%	1.020%	1.017%	1.017%
SPD-06463	5	A	17	0.971%	0.968%	0.965%	0.964%	0.973%	0.970%	0.969%	0.967%	0.972%	0.969%	0.967%	0.966%
		B	18	0.971%	0.966%	0.962%	0.962%	0.972%	0.969%	0.968%	0.966%	0.970%	0.968%	0.966%	0.965%
		C	19	0.971%	0.968%	0.964%	0.964%	0.973%	0.970%	0.969%	0.967%	0.972%	0.969%	0.967%	0.967%
		D	20	0.967%	0.964%	0.960%	0.960%	0.969%	0.966%	0.964%	0.963%	0.968%	0.966%	0.964%	0.963%
SPD-06464	6	A	21	1.023%	1.019%	1.016%	1.015%	1.025%	1.022%	1.020%	1.019%	1.024%	1.021%	1.019%	1.018%
		B	22	1.022%	1.017%	1.013%	1.013%	1.023%	1.019%	1.018%	1.016%	1.021%	1.018%	1.017%	1.015%
		C	23	1.020%	1.016%	1.012%	1.012%	1.021%	1.019%	1.017%	1.016%	1.020%	1.018%	1.016%	1.015%
		D	24	1.018%	1.014%	1.010%	1.010%	1.020%	1.017%	1.015%	1.013%	1.018%	1.017%	1.014%	1.013%
SPD-06466	7	A	25	1.030%	1.026%	1.022%	1.022%	1.031%	1.029%	1.027%	1.026%	1.030%	1.027%	1.025%	1.024%
		B	26	1.028%	1.024%	1.021%	1.021%	1.029%	1.027%	1.025%	1.023%	1.028%	1.026%	1.024%	1.023%
		C	27	1.028%	1.024%	1.021%	1.021%	1.029%	1.027%	1.026%	1.024%	1.028%	1.027%	1.024%	1.023%
		D	28	1.029%	1.026%	1.022%	1.022%	1.031%	1.028%	1.026%	1.024%	1.030%	1.028%	1.026%	1.024%
SPD-06468	8	A	29	1.029%	1.025%	1.021%	1.021%	1.031%	1.028%	1.027%	1.025%	1.029%	1.026%	1.024%	1.024%
		B	30	1.030%	1.026%	1.022%	1.022%	1.031%	1.028%	1.027%	1.025%	1.030%	1.028%	1.026%	1.025%
		C	31	1.028%	1.024%	1.021%	1.021%	1.030%	1.027%	1.025%	1.023%	1.029%	1.027%	1.024%	1.023%
		D	32	1.028%	1.024%	1.021%	1.021%	1.029%	1.027%	1.025%	1.023%	1.028%	1.026%	1.024%	1.023%
			Mean:	1.019%	1.015%	1.012%	1.012%	1.020%	1.018%	1.016%	1.014%	1.019%	1.017%	1.015%	1.014%
			Min:	0.967%	0.964%	0.960%	0.960%	0.969%	0.966%	0.964%	0.963%	0.968%	0.966%	0.964%	0.963%
			Max:	1.032%	1.031%	1.024%	1.024%	1.033%	1.031%	1.029%	1.028%	1.032%	1.030%	1.027%	1.027%
